# The Association between Galectin-3 and hs-CRP and the Clinical Outcome after Non-ST-Elevation Myocardial Infarction with Preexisting Atrial Fibrillation

**DOI:** 10.1038/s41598-017-15265-0

**Published:** 2017-11-08

**Authors:** Milan Pavlović, Svetlana Apostolović, Dragana Stokanović, Stefan Momčilović, Tatjana Jevtović-Stoimenov, Snezana Ćirić Zdravković, Sonja Šalinger Martinović, Nebojsa Krstić, Goran Koraćević, Danijela Djordjevic, Vladan Ćosić, Valentina N. Nikolic

**Affiliations:** 1 Department of Internal Medicine — Cardiology, Medical Faculty, University of Nis, Bulevar dr Zorana Djindjica 81, Nis, Serbia; 2 Clinic for Cardiovascular Diseases, Clinical Centre Nis, Bulevar dr Zorana Djindjica 48, Nis, Serbia; 3 Department of Pharmacology and Toxicology, Medical Faculty, University of Nis, Bulevar dr Zorana Djindjica 81, Nis, Serbia; 4 Medical Faculty, University of Nis, Bulevar dr Zorana Djindjica 81, Nis, Serbia; 5 Institute of Biochemistry, Medical Faculty, University of Nis, Bulevar dr Zorana Djindjica 81, Nis, Serbia; 6 Medical Biochemistry Center, Clinical Centre Nis, Bulevar dr Zorana Djindjica 48, Nis, Serbia

## Abstract

Increased galectin-3 plasma concentration has been linked to an unfavorable outcome in patients with heart failure or atrial fibrillation (AF). There are no published data about the prognostic utility of galectin-3 and high-sensitivity C-reactive protein (hs-CRP) for long-term clinical outcome in the Non-ST elevation acute myocardial infarction (NSTEMI) patients with preexisting AF. Thirty-two patients with the first acute NSTEMI and preexisting AF and 22 patients without preexisting AF, were prospectively followed for fifteen months. Patients with AF had significantly higher galectin-3 plasma levels (p < 0.05) and hs-CRP concentration (p < 0.01), compared with patients without AF. Galectin-3 plasma concentration was not a significant covariate of the composite outcomes (p = 0.913). Patients with high hs-CRP (above 4.55 mg/L) showed 2.5 times increased risk (p < 0.05) of the composite outcome occurrence (p < 0.05). Besides, three-vessel coronary artery disease, creatinine serum level, and creatinine clearance were significant covariates (p < 0.05; p < 0.05; p < 0.01) of the composite outcome, respectively. Creatinine clearance, solely, has been shown to be an independent predictor of unfavorable prognosis after a 15-month follow-up. Galectin-3 and hs-CRP plasma levels were elevated in NSTEMI patients with AF, but with differential predictive value for an unfavorable clinical outcome. Only hs-CRP was associated with increased risk of composite outcome occurrence.

## Introduction

Atrial fibrillation (AF) is a common cardiac arrhythmia showing an increase in prevalence due to an increased age of the population, more successful treatment of cardiovascular diseases and mortality reduction^1^. Patients with atrial fibrillation are at an increased risk of developing a serious complication, including stroke, heart failure, dementia and an early mortality^2^. Ischemic heart disease and acute coronary syndromes are leading causes of death worldwide, and an estimate is that it will maintain such position in the years to come^3–5^. Reported frequency of preexisting AF in the setting of acute myocardial infarction ranges from 1–13%^6^. When these two disorders are associated, both short-term and long-term patients’ prognosis are unfavorable^7–10^.

In studies of patients with AF, increased galectin-3 plasma levels have been found, compared with the patients without AF^11,12^. A biomarker of fibrosis, plasma galectin-3, has been shown to be a significant predictor of left atrial and left ventricular remodeling, as well as of left ventricular dysfunction^13,14^, and also of an increased risk of developing complications of AF^15^. Numerous studies of heart failure have shown the prognostic significance of the increased galectin-3 plasma concentration, in the prediction of an unfavorable outcome^16,17^. Unlike for heart failure in the acute myocardial infarction, there is less data for the role of galectin-3 as a predictor of clinical outcome. There are no published data about a relation of galectin-3 and high-sensitivity C-reactive protein (hs-CRP) plasma levels in the Non-ST elevation acute myocardial infarction (NSTEMI) patients with preexisting AF and long-term clinical outcome.

The goal of this study is to assess the impact of AF on galectin-3 plasma level in patients hospitalized for a first NSTEMI, without reduced left ventricular ejection fraction (LVEF) and signs of heart failure. The relation between galectin-3 plasma level in these patients and their clinical outcomes after 15-month follow-up was also studied. The impact of preexisting AF on the biomarker of inflammation hs-CRP in these patients was also evaluated, as well as the impact of hs-CRP on prognosis after a 15-month follow-up.

## Results

### Population

The average age of the patients was 68.1 ± 10.9 years (ranged from 43 to 91 years), and 32 men and 22 women were included in the study. Baseline characteristics of the patients’ groups hospitalized for the first acute NSTEMI, without findings of LVEF and signs of heart failure, with AF and without AF are presented in Table 1. A statistically significant difference in age, sex, or other demographic characteristics, as well as in the rate of arterial hypertension and diabetes mellitus, between the groups of patients with and without AF, was not found. A significant difference in the high-sensitivity troponin I, B-type natriuretic peptide, or in the number of coronary arteries with a significant angiographic stenosis, or difference in the rate of significant stenosis in the proximal and/or medial segment of the left anterior descending artery (LAD), between the groups of patients with and without AF, was not found. Ejection fraction was not significantly different between patients with AF 54.1 ± 15.3% and without AF 57.1 ± 8.6%. Patients with AF had significantly higher left ventricular end-diastolic index (p < 0.05), and greater left atrial volume index, but without statistical significance. There were no significant differences in the rate of myocardial revascularization (percutaneous coronary intervention and coronary artery by-pass graft) during the acute NSTEMI treatment, as well as in the frequency of use of medication for secondary prevention of coronary artery disease. Patients with AF were taking a chronic oral anticoagulant therapy and antiarrhythmic drug therapy following recommendations from the current European Sociaty of Cardiology (ESC) and American College of Cardiology/American Heart Association (ACC/AHA) guidelines.

**Table 1:** Baseline characteristics of the patients with and without AF.

	with AF (N = 32)	without AF (N = 22)	*t (p) or **Z (p) or ***χ^2^ (p)
Age (years)	68.81 ± 9.48	67.14 ± 12.82	0.523 (0.604)*
Gender (female)	15 (46.9%)	7 (31.8%)	0.680 (0.410)***
Body-mass index (kg/m^2^)	25.94 ± 4.88	25.72 ± 3.34	0.192 (0.849)*
Smoking	13 (40.6%)	9 (40.9%)	0.000 (1.000)***
Arterial hypertension	31. (96.9%)	19 (86.4%)	0.847 (0.357)***
Diabetes mellitus	6 (18.8%)	9 (40.9%)	2.182 (0.140)***
High-sensitivity troponin I (ng/l)	4.16 ± 14.38	2.16 ± 3.16	0.492 (0.623)**
B-type natriuretic peptide (pg/ml)	385.35 ± 502.73	321.92 ± 512.45	1.259 (0.208)**
Coronary artery disease (multi-vessel)	11 (55.0%)	10 (55.6%)	0.000 (1.000)***
LAD proximal and/or medial stenosis	12 (60.0%)	9 (52.9%)	0.085 (0.770)***
left ventricular end-diastolic volume index (ml/m^2^)	42.29 ± 11.70	34.15 ± 12.05	2.045 (0.048)*
left ventricular end-systolic volume index (ml/m^2^)	19.48 ± 8.65	14.93 ± 7.32	1.653 (0.107)*
LVEF (%)	55.25 ± 10.16	54.94 ± 6.92	0.110 (0.913)*
left atrial volume index (ml/m^2^)	48.17 ± 28.29	38.17 ± 28.29	0.710 (0.478)**
hs-CRP (mg/l)	21.66 ± 26.04	5.47 ± 6.06	2.910 (0.004)**
Galectin-3 (ng/ml)	10.01 ± 2.49	8.57 ± 2.50	2.088 (0.042)*
Creatinine clearence (ml/min.)	59.48 ± 29.01	62.55 ± 24.63	0.406 (0.687)*
Cholesterol (mmol/l)	5.18 ± 1.55	5.15 ± 1.41	0.255 (0.799)**
LDL (mmol/l)	3.24 ± 1.03	3.28 ± 1.26	0.129 (0.898)*
HDL (mmol/l)	1.05 ± 0.33	1.06 ± 0.28	0.061 (0.951)*
Triglycerides (mmol/l)	1.78 ± 1.55	2.39 ± 3.61	0.590 (0.555)**
Percutaneous coronary intervention	15 (46.9%)	11 (50.0%)	0.003 (0.956)***
Coronary artery by-pass graft	6 (18.8%)	5 (22.7%)	0.000 (1.000)***
Beta-blocker	32 (100.0%)	22 (100.0%)	
ACE-inhibitor/Angiotensine receptor antagonist	23 (71.9%)	13 (59.1%)	0.470 (0.493)***
Statin	25 (80.6%)	21 (95.5%)	1.340 (0.247)***
Acetylsalycilic acid	31 (96.9%)	22 (100.0%)	1.340 (0.247)***
Ticagrelor/clopidogrel	32 (100.0%)	22 (100.0%)	

### Galectin-3 plasma concentrations

Galectin-3 plasma concentration in patients with the first acute NSTEMI, without reduced LVEF and signs of heart failure, ranged from 3.82 up to 14.10 ng/ml (mean value 9.42 ± 2.57). Patients with AF had statistically significant higher values of galectin-3 plasma levels 10.01 ± 2.49 ng/ml, compared with patients without AF 8.57 ± 2.50 (p < 0.05). A statistically significant difference between galectin-3 plasma levels in patients with paroxysmal AF and patients with permanent/persistent AF was not found (t = 0.358, p = 0.723). A statistically significant correlation between galectin-3 plasma levels and studied demographic parameters, left ventricular and atrial volume indices, biomarkers high-sensitivity troponin I, hs-CRP, and B-type natriuretic peptide, or biochemical tests results, was not found. Also, a significant difference in galectin-3 plasma levels between patients categorized according to different category variables was not found (smoking status, co-morbidities such as diabetes mellitus and arterial hypertension, preserved/mid-range LVEF, multi-vessel coronary artery disease, LAD proximal and/or medial stenosis), except for the higher values of galectin-3 in female patients (10.27 ± 2.53 ng/ml vs. 8.83 ± 2.47 ng/ml, t = 2.080, p < 0.05). After performing a series of univariate linear regression modeling, the only independent factor that stood up as a significant covariate of galectin-3 plasma level was the presence of AF (t = 2.088, p = 0.042). The receiver operating characteristic curve analysis was done to determine the optimal galectin-3 concentration cut-off value for prediction of AF in patients with first acute NSTEMI, without reduced LVEF and signs of heart failure. The optimal cut-off value was estimated at 7.53 ng/ml (AUC = 0.661, 95%CI: 0.511–0.810, sensitivity 90.6%, specificity 45.5%, p < 0.05).

Considering the cut-off value of galectin-3 plasma levels, a comparison between characteristics of the patients with high galectin-3 plasma levels (>7.53 ng/ml) and low plasma levels (<7.53 ng/ml) was made. In the group of patients hospitalized for a first acute NSTEMI, without reduced LVEF, and with high galectin-3 plasma levels, there were significantly more patients with AF (70.7%), compared with the group with low galectin-3 plasma levels (23.1%, p < 0.01). There were also significantly more patients with permanent/persistent AF (34.1%) in the group with high galectin-3 plasma levels, compared with the group with low galectin-3 plasma levels (15.4%, p < 0.01). Patients with high galectin-3 plasma levels were not significantly different, considering demographic characteristics and co-morbidity, from the patients with low galectin-3 plasma levels. On the other hand, in the group with high galectin-3 plasma level, there was a trend that women compared to men are older, but without statistical significance (70.83 ± 8.75 years vs. 64.87 ± 10.43 years, p = 0.059). A significant difference in the biomarker of necrosis high-sensitivity troponin I concentration, or in the biomarker of hemodynamic stress, B-type natriuretic peptide concentration, between the groups with high and low galectin-3 plasma levels, was not found. A significant difference in a number of coronary arteries with a significant angiographic stenosis, as well as in the rates of coronary stenosis localized in the proximal and/or medial segment of the left anterior descending artery, between the groups with high and low galectin-3 plasma levels, was not found. A significant difference in LVEF, left ventricular end-diastolic and end-systolic volume indices and left atrial volume index between the patients’ groups with high and low galectin-3 plasma levels, was not found. Patients with high galectin-3 plasma levels had significantly higher concentrations of hs-CRP (18.43 ± 24.22 mg/l), compared with patients with low galectin-3 levels (5.43 ± 6.73 mg/l, t = 2.285, p < 0.05). A significant difference in rate and modality of myocardial revascularization (percutaneous coronary intervention and coronary artery by-pass graft), as well as in pharmacotherapy, between the groups with high and low galectin-3 levels, was not found.

Univariable binary logistic regression analysis was performed to identify the independent variables that may predict the preexistence of AF in patients with first acute NSTEMI, without reduced LVEF and signs of heart failure. Statistically significant models were obtained with the following variables: galectin-3 concentration, galectin-3 plasma level above 7.53 ng/ml and hs-CRP concentration (Table 2). High galectin-3 concentration is associated with more than 8-fold probability of AF in these patients, and it may be accounted for 15.9–21.5% of the variance in AF preexistence. It has higher predictive value than the concentration of galectin-3. A similar impact was found for the level of hs-CRP as an independent variable. With an increase of hs-CRP concentration, for each milligram per liter, the probability increases 1.1 times. These variables were included in the multivariable analysis. A statistically significant model (χ^2^ = 17.598, p < 0.001) was obtained. These variables combined may explain 28.3–38.2% of the variance in AF presence in patients with first acute NSTEMI, without reduced LVEF and signs of heart failure. In this multivariable model, high galectin-3 level (above the previously determined threshold), was the only variable found to be an independent predictor of AF, associated with a 10-fold probability of AF (p < 0.05).

**Table 2:** Univariable binary logistic regression analysis of AF preexistence, as a dependent variable, in patients with NSTEMI without reduced left ventricular ejection fraction.

	Cox and Snell R^2^	Nagelkerke R^2^	B	SE	Wald	p	HR	HR 95% CI
Galectin-3 (ng/ml)	0.077	0.104	0.237	0.120	3.922	**0.048**	**1.267**	**1.002–1.602**
High galectin-3 level (>7.53 ng/ml)	0.159	0.215	2.086	0.742	7.898	**0.005**	**8.056**	**1.880–34.516**
hs-CRP (mg/l)	0.190	0.257	0.093	0.044	4.551	**0.033**	**1.097**	**1.008–1.195**

### High sensitivity-CRP plasma concentrations

Patients with AF, hospitalized for the first acute NSTEMI, without reduced LVEF and signs of heart failure, had a significantly higher level of the biomarker of inflammation, hs-CRP, 21.66 ± 26.04 mg/l, compared with patients without AF (5.47 ± 6.06 mg/l, p < 0.01) (Table 1). A significant relation between hs-CRP concentration and the marker of myocardial necrosis, hs-TnI concentration, coronary artery disease extensiveness relative to the number of coronary arteries with the significant angiographic stenosis, as well as the existence of a significant stenosis in the proximal and/or medial segment of the left anterior descending coronary artery, was not found. A significant negative correlation was found between hs-CRP level and LVEF (r = −0.334, p < 0.05). A significant correlation between hs-CRP level and the left ventricular end-diastolic and end-systolic volume indices, was not found. A significant correlation was found between hs-CRP levels and metabolic parameters: serum urea (r = 0.355, p < 0.01), serum creatinine levels (r = 0.421, p < 0.01) and creatinine clearance (r = 0.420, p < 0.01).Using receiver operating characteristic curve analysis, a cut-off value hs-CRP concentration was determined, depending on the AF status. It was estimated at 4.55 mg/l (AUC = 0.738, 95%CI: 0.605–0.871, sensitivity 71.9%, specificity 71.4%, p < 0.01).

### Follow up

**Figure 1:**
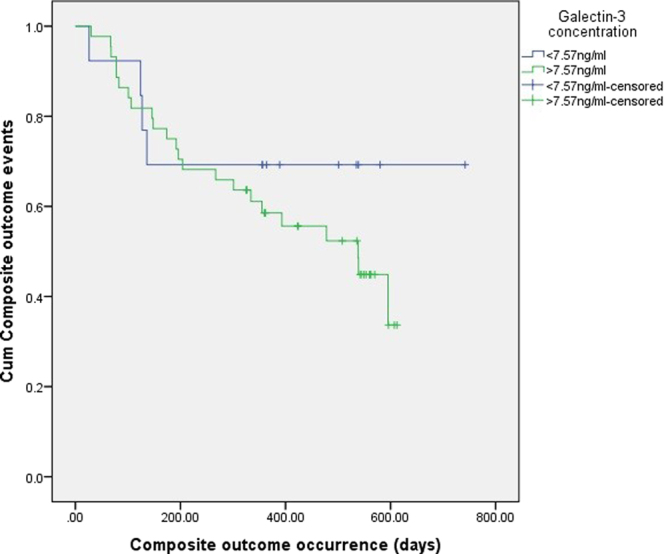
It can be seen from the Kaplan-Meier curve that the previously determined cut-off value for galectin-3 concentration was not significant factor predicting composite outcome occurrence in patients (χ^2^ = 0.881, p = 0.348).

Follow-up of the patients hospitalized for a first acute NSTEMI, without reduced LVEF and signs of heart failure, was 461.3 ± 166.4 days. During this time, seven patients (13.0%) have died from cardiovascular causes. The composite of hard events, defined as mortality from cardiovascular causes, nonfatal myocardial infarction, or nonfatal stroke, was observed in 16 patients (29.6%). Nine patients had a nonfatal myocardial infarction, and there were no nonfatal strokes during follow-up. Composite outcome of mortality from cardiovascular causes, nonfatal myocardial infarction, nonfatal stroke or hospitalization for heart failure or urgent myocardial revascularization was registered in 27 patients (50.0%). Four patients had hospitalization for heart failure and seven patients for urgent myocardial revascularization. There was no statistically significant difference in the frequency of mortality from cardiovascular causes (χ^2^ = 0.865, p = 0.352), composite of hard events (χ^2^ = 0.058, p = 0.810) and composite outcome (χ^2^ = 0.000, p = 1.000), between patients with and without AF. Using Cox-regression analysis, we have not observed the significance of the galectin-3 level for the mortality from cardiovascular causes (χ^2^ = 2.757, p = 0.097). Galectin-3 concentration was not a significant covariate of composite hard endpoints occurrence (χ^2^ = 0.061, p = 0.806), nor composite outcome (χ^2^ = 0.012, p = 0.913). Previously determined cut-off value for galectin-3 concentration, estimated at 7.53 ng/ml, was not significant factor predicting mortality from cardiovascular causes, (χ^2^ = 0.376, p = 0.540), composite of hard endpoints (χ^2^ = 0.034, p = 0.853), nor composite outcome (χ^2^ = 0.001, p = 0.978) (Fig. 1).

**Figure 2:**
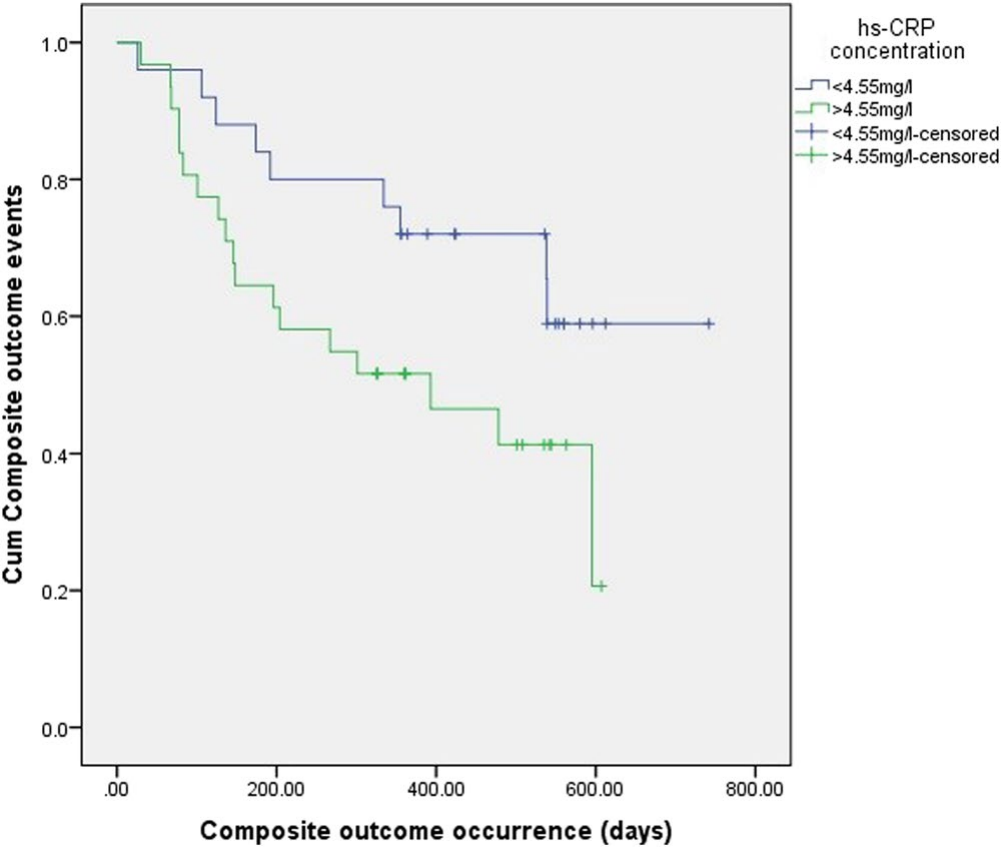
It can be seen from the Kaplan-Meier curve that the previously determined cut-off value for hs-CRP concentration was significant factor predicting composite outcome occurrence in patients (χ^2^ = 4.082, p < 0.05).

Patients with high hs-CRP (above previously determined cut-off at 4.55 mg/l) showed 2.5 times increased risk (p < 0.05) of composite outcome occurrence (χ^2^ = 4.802, p < 0.05) (Fig. 2). Three-vessel coronary artery disease, as a significant covariate of composite endpoints occurrence (χ^2^ = 5.799, p < 0.05), increases the risk 3.3 times (p < 0.05). Besides, creatinine level was a significant covariate (χ^2^ = 4.695, p < 0.05), as well as creatinine clearance (χ^2^ = 8.619, p < 0.01), of composite outcome. In patients with AF (χ^2^ = 5.158, p < 0.05), this risk rises to 4.6 (p < 0.05). Diabetic patients had almost 5 times higher risk of composite end-points (p < 0.05). After performing a series of univariate logistic regression modeling with the composite outcome as a dependent variable, 3-vessel coronary artery disease, diabetes mellitus, and creatinine clearance were statistically significant covariates. Creatinine clearance remained independently significant: the increase in creatinine clearance for 1 ml/min decreases the risk 1.1 times.

## Discussion

In our study, galectin-3 plasma concentration in patients with the first acute NSTEMI, without reduced LVEF and signs of heart failure, was significantly higher in the group of patients with preexisting AF, compared with the patients without AF. Galectin-3 plasma level was not significantly different between patients with paroxysmal AF and those with permanent/persistent AF. We have not found a significant correlation between galectin-3 plasma concentration and the marker of myocardial necrosis high-sensitivity troponin I concentration, nor an association between galectin-3 plasma levels and a number of coronary arteries with significant angiographic stenosis. So far, data about the impact of preexisting atrial fibrillation on galectin-3 plasma levels in the patients with acute coronary syndromes were not published. In other studies, a higher galectin-3 concentration was found in the patients with AF and preserved LVEF, compared with the patients without AF^11,12^. Higher levels of the biomarker of fibrosis galectin-3 were found in the patients with permanent and persistent AF, compared with the patients with paroxysmal atrial fibrillation^12,13^. Adding galectin-3 to CHA2DS2-VAScscore has increased the predictivity of the risk stratification in the patients with AF^13^. It has been shown that higher galectin-3 plasma level was related with a higher risk of AF occurrence in the next ten years^15^. Subjects were classified into four groups relative to quartile values of galectin-3 plasma concentration during the initial examination. Patients in the quartile with the highest galectin-3 plasma concentration have shown the highest risk of a late AF occurrence. After adjustments for traditional risk factors for AF, relation with galectin-3 plasma levels lost its significance^15^. In an acute myocardial infarction, myocytes necrosis and an increased stretching of the infarcted ventricular wall segment, stimulate the production of galectin-3 by macrophages in the process of affected ventricular wall reparation. In the later course of myocardial infarction evolution, chronic profibrotic activation may result in a progression of left ventricular remodeling and development of systolic dysfunction^18^.

In our study, galectin-3 plasma concentration, determined on the third day of hospitalization for the first acute NSTEMI, without reduced LVEF and signs of heart failure, has not shown a significant correlation with LVEF, left ventricular end-diastolic and end-systolic volume indices and left atrial volume index, which were determined on the first day of hospitalization using echocardiography, nor with the BNP plasma concentration. So far, data about a relation between galectin-3 plasma levels in the patients with acute coronary syndromes and preexisting AF and LVEF, left ventricular end-diastolic and end-systolic volume indices and left atrial volume index, were not published. In some recent studies, galectin-3 plasma concentration has been shown to be a significant predictor of left ventricular remodeling, as well as of dysfunction of the left ventricle in the patients with AF^13,14^. It has been demonstrated that patients with a permanent and persistent AF had more prominent myocardial fibrosis, which was evaluated by cardiac magnetic resonance imaging using late gadolinium enhancement^14^. Galectin-3 maintained a significant predictivity for reduced LVEF, even after adjusting for factors that may have the impact on ventricular dysfunction development^19^. The correlation between galectin-3 plasma level and left atrial volume, which is an indicator for left atrial remodeling, was found^11^. In a study that included patients within the first two weeks of an acute myocardial infarction, with a reduced LVEF < 40%, evaluated by echocardiography, but without signs of heart failure, a significant correlation between galectin-3 plasma concentration and LVEF, left ventricular end-diastolic and end-systolic volume indices determined using cardiac magnetic resonance during the baseline hospitalization, was not demonstrated^20^. The baseline value of galectin-3 has shown a significant negative correlation with LVEF on the repeated cardiac magnetic resonance after 24 weeks, but the correlation with left ventricular end-diastolic and end-systolic volume indices has not been shown. It has been demonstrated in the group of 247 patients with a first ST-elevation myocardial infarction treated with primary percutaneous coronary intervention, that there was a significant association between galectin-3 plasma concentration, determined on the third day of hospitalization, and LVEF, infarct size and left ventricular end-systolic volume index, which were determined after four months using cardiac magnetic resonance^21^. Patients with higher baseline values of galectin-3 had significantly lower LVEF after four months, compared with the patients with lower baseline values of galectin-3. Repeated sample of galectin-3, taken after four months, at the time of the cardiac magnetic resonance examination, did not show a significant association with LVEF and infarct size.

In our study, hs-CRP plasma concentration in the patients hospitalized for a first acute NSTEMI, without reduced LVEF and signs of heart failure, was significantly higher in the group of patients with atrial fibrillation, compared with the patients without AF. A significant association between hs-CRP plasma concentration and the marker of myocardial necrosis, high-sensitivity troponin I concentration, has not been found, nor with a number of coronary arteries with significant angiographic stenosis. There is no published data on the impact of preexisting AF on hs-CRP plasma levels in the patients with acute coronary syndromes. A substantial inflammatory response is an integral component of the response to tissue injury during an acute myocardial infarction. In a large cohort of patients with acute myocardial infarction, there was a graded positive association between increased CRP and new-onset AF^22^. It has been demonstrated in studies that an increased hs-CRP plasma concentration was related to an increased risk of AF^23–25^ and coronary artery disease in male patients^25^. It is not known whether inflammation precedes the occurrence of AF or it is its consequence, or simply coexists with AF^26^. We have not found the presence of a significant correlation between hs-CRP plasma levels and left ventricular end-diastolic and end-systolic volume indices and left atrial volume index. Furthermore, in our cohort, the patients with higher hs-CRP plasma levels had a lower LVEF.

The present study did not show significant association between preexisting AF and clinical outcome after 15-month follow-up the patients hospitalized for a first acute NSTEMI without reduced LVEF and signs of heart failure. In contrast to these findings, the several studies have found that patients with preexisting AF had significantly higher total mortality, cardiovascular death, stroke, and all-cause mortality after an acute myocardial infarction^7,8,27–29^. There is no published data on the prognostic significance of galectin-3 plasma levels in the patients hospitalized for acute coronary syndromes with preexisting AF. Previous publications have suggested that galectin-3 can be viewed as a useful predictor for new-onset AF during hospitalization due to acute myocardial infarction, as well as to later occurrence of AF, during follow-up. In 145 consecutive patients with a first myocardial infarction, treated with PCI, Szadkowska *et al*. demonstrated that increased galectin − 3 levels were independently associated with more frequently occurrence of a new-onset atrial fibrillation, as well as with more frequently administered diuretic treatment during hospitalization^30^. Galectin - 3 levels were determined on 3rd to 5th hospitalization day and patients with preexisting heart failure were not included. There were more women, elderly patients, patients with diabetes mellitus, renal dysfunction, and patients with preexisting chronic (permanent) atrial fibrillation in the group of patients with increased galectin - 3 levels. In the same cohort, 49 patients with non-STEMI were studied. The authors did not compared Gal-3 levels in NSTEMI and STEMI patients. Tunon *et al*. performed study with 706 patients with coronary disease which have been followed-up over the period of 2.2 years^31^. An independent relation between increased galectin - 3 levels and mortality and/or heart failure was found. Galectin-3 and atrial fibrillation showed an independent predictive value of composite clinical outcome of mortality, nonfatal myocardial infarction, nonfatal stroke, transient ischemic attack and/or heart failure. Elderly patients with coronary artery disease had a higher rate of acute ischemic events. Biomarker of inflammation, hs-CRP, has not shown a significant relation with clinical outcome.

A significant association between an increased galectin-3 plasma concentration and unfavorable short-term^16^ and long-term clinical outcome^17^ has been demonstrated in the patients with heart failure. It has been shown the even higher predictive significance of galectin-3 plasma levels in the patients with heart failure and preserved LVEF^17,32^. Measurements of galectin-3 plasma levels have recently entered AHA recommendation for stratification of heart failure risk. Unlike for heart failure in the acute myocardial infarction, there is less data for the role of galectin-3 as a predictor of unfavorable clinical outcome.

The significance of galectin-3 in atherogenesis has been shown in both *in vivo* and *in vitro* studies^26^. An increased galectin-3 level was associated with a more intense vascular inflammation and a higher migration of monocytes into the arterial walls, as well as later transformation of macrophages into foam cells. Along with progression of atheromas, the significance of galectin-3 has been demonstrated in atheromas destabilization, and atherothrombosis development. A predictive significance of galectin-3 in long-term cardiovascular mortality in the high-risk patients with coronary disease has been demonstrated^33^. In a study with patients with acute myocardial infarction treated with PCI, an independent predictive value of galectin-3 has been demonstrated in reinfarction occurence, early after the first myocardial infarction^34^.

Furthermore, in the present study, increased hs-CRP plasma levels, which have shown a significant relation with preexisting AF, were associated with unfavorable prognosis after a 15-month follow-up. Patients with an increased hs-CRP showed a significantly higher risk of the composite outcome of mortality from cardiovascular causes, nonfatal myocardial infarction, nonfatal stroke or hospitalization for heart failure or urgent myocardial revascularization occurrence. There is no published data on the prognostic significance of hs-CRP plasma levels in the patients hospitalized for NSTEMI with preexisting AF. In another group of 346 patients with ST-elevation myocardial infarction, increased hs-CRP levels, as well as oxidative stress markers levels, has been reported to be a useful predictor for more often occurrence of atrial fibrillation in the acute phase of a myocardial infarction^35^. Hs-CRP measured after stabilization post-ACS strongly predicts an outcome after 3 to 12 months^36^. It was found that elevated levels of CRP had been related to patient instability and an increased risk of death, myocardial infarction, and the need for urgent revascularization. In some recent studies have been shown that patients with atrial fibrillation and higher hs-CRP levels had increased risk of ischemic stroke and mortality^25,37,38^. Of interest, addition of hs-CRP in the risk assessment for the patients with AF may increase prognostic predictivity of the CHA2DS2-VASc score^39^.

In current study, the presence of three-vessel coronary disease, diabetes mellitus, and reduced creatinine clearance during hospitalization for an acute NSTEMI was associated with an unfavorable prognosis after 15-months and significantly more frequent development of composite outcome. From studied factors, only creatinine clearance has shown an independent predictability of unfavorable prognosis after a 15-month follow-up. It has been demonstrated^40^ that risk of recurrent ischemic events was more dependent on the presence of multifocal lesions other than the culprit lesion responsible for the acute coronary syndrome event. The percentage of patients with more than one active plaque on angiography was related to an increasing baseline level of the marker of inflammation, C-reactive protein. It has been shown that chronic kidney disease was associated with worse outcomes following ACS. Chronic kidney disease represented an independent risk factor for death and recurrent cardiovascular events following acute coronary syndrome^41^.

Finally, high galectin-3 plasma levels have shown a significant and independent predictive value of recurrence after RF ablation for atrial fibrillation^42^. Over one-year follow-up, the patients with a lower both Galectin-3 levels (<15 ng/ml) and left atrium diameter (less than 40 mm) initially, had a 1-year better arrhythmia-free survival rate of 91%, after a single procedure and without administration of an arrhythmic drug.

Although follow-up in our study was 15 months, the association of the studied biomarkers with clinical outcome following NSTEMI should be considered carefully, because of the number of the patients in the study that represents a limitation factor for evaluation of the prognostic significance. But these patients were consecutive patients with preexisting atrial fibrillation, hospitalized over the period of 14 months when a total of 660 patients were hospitalized for a first acute myocardial infarction. We measured galectin-3 only once at baseline, on the third day after acute myocardial infarction. It is not known whether galectin-3 levels fluctuate in plasma early after acute myocardial infarction, or during the 15-months period that served as a follow-up in our study. Our findings apply to patients with NSTEMI, without reduced LVEF on echocardiography performed within 24 hours of infarction, and without signs of heart failure, and, therefore, cannot be extrapolated to all patients with acute myocardial infarction.

We found that galectin-3 and hs-CRP plasma concentrations were significantly higher in the group of patients with preexisting AF, compared with patients without AF. The only independent factor that stood up as a significant covariate of galectin-3 plasma level was the presence of AF. Atrial fibrillation has not shown significant association with clinical outcome after a 15-month follow-up. No significant association was found between galectin-3 plasma level and an unfavorable outcome. Patients with an increased hs-CRP showed a significantly higher risk of the composite outcome of mortality from cardiovascular causes, nonfatal myocardial infarction, nonfatal stroke or hospitalization for heart failure or urgent myocardial revascularization occurrence. Besides, three-vessel coronary artery disease, creatinine serum level, and creatinine clearance were significant covariates of the composite outcome, respectively. Only creatinine clearance has shown an independent predictability of unfavorable prognosis after a 15-month follow-up.

To the best of our knowledge, this study is the first to report that elevated galectin-3 and hs-CRP plasma levels in NSTEMI patients with preexisting AF with differential predictive value for an unfavorable clinical, long-term outcome. Further studies in larger and independent series should be carried out to confirm these our findings.

## Methods

Thirty-two consecutive patients with preexisting atrial fibrillation with duration of 12.2 ± 4.1 months were enrolled in the study. They were hospitalized for a first NSTEMI, without findings of reduced LVEF, which was assessed by echocardiography at admission (LVEF ≥ 40%). These patients were admitted to University Clinic for Cardiovascular Diseases, Clinical Center Nis, Serbia, over the period of 14 months, when a total of 660 patients was hospitalized for a first acute myocardial infarction. 14 patients had permanent or persistent AF with an average duration of 13.4 ± 4.3 months before hospitalization for NSTEMI, and 18 patients had paroxysmal AF with duration of 10.9 ± 3.5 months before admission. 22 consecutive patients without preexisting atrial fibrillation were also included in the study, and they were hospitalized for a first NSTEMI, without findings of reduced LVEF at admission. Both groups were similar in age (mean 67 ± 11.18 years, range 42–91 years) and gender structure (female 61.4%). Only patients who had preserved LVEF ≥ 50% or mid-range LVEF 40–49%^43^ at the initial echocardiography examination were included in the study. Patients with signs of heart failure or with previously diagnosed heart failure have been excluded from the study. Non-invasive and invasive diagnostics, pharmacotherapy, as well as percutaneous or surgical myocardial revascularization were performed following the institution doctrine and the current European Society of Cardiology (ESC) and American College of Cardiology/American Heart Association (ACC/AHA) guidelines. The informed consent has been obtained from all the patients before their inclusion in the study. The research was performed following the principles highlighted in 1975 Helsinki Declaration and approved by the Ethics Committee of the Faculty of Medicine, University of Nis, Nis, Serbia

Transthoracic echocardiography was performed on the first day of hospitalization for an NSTEMI. The volume of the left ventricle at the end of diastole and the end of systole, and the volume of the left atrium at the end of systole were determined by biplane planimetry using Simpson’s formula; LVEF, left ventricular end-diastolic and end-systolic volume indices and left atrial volume index were calculated. On the first day of hospitalization, biochemical and hematology tests, high-sensitivity troponin I, high-sensitivity C-reactive protein (hs-CRP) and B-type natriuretic peptide tests were performed. Blood sample for galectin-3 determination was taken on the third day. Plasma was separated from whole blood by centrifugation at the temperature of 25 °C for 10 min. at 3000 g and stored at −80 °C for subsequent analysis. Commercially available enzyme-linked immunosorbent assays (ELISA) were used, according to manufacturer’s instructions, for the determination of galectin-3 (BG Medicine, Inc. Waltham, MA, USA), high-sensitivity troponin I, hs-CRP and B-type natriuretic peptide plasma levels.

The clinical outcome after 15-month follow-up was evaluated and mortality from cardiovascular causes, composite of hard events defined as mortality from cardiovascular causes, nonfatal myocardial infarction, or nonfatal stroke and the composite outcome of mortality from cardiovascular causes, nonfatal myocardial infarction, nonfatal stroke or hospitalization for heart failure or urgent myocardial revascularization were registered.

We used Statistical Package for Social Sciences (SPSS 21.0; Chicago, IL, USA) for data analysis. Baseline characteristics are presented as frequencies or means with SDs. Both parametric methods (Student’s t-test), for quantitative variables, and non-parametric methods (Mann-Whitney U-test) were used. Pearson’s correlation coefficient was used as a measure of linear relationship. Fisher’s exact test was performed to determine the association of qualitative variables. Significant predictors of the dependent variable variance were identified by standard linear, univariate and multivariate, or binary logistic regression modeling. Besides, the receiver operating characteristic curve analyses, for the optimal cut-off value of galectin-3 and hs-CRP determination, was performed. Kaplan-Meier survival analysis was performed in order to determine significant variables for the clinical outcomes. A p-value less than 0.05 was considered to be a measure of statistical significance.

The datasets generated during and/or analyzed during the current study are available from the corresponding author on reasonable request.

## Electronic supplementary material

Dateset 1
